# Giant Retroperitoneal Schwannoma: Case Report and Review of the Literature

**DOI:** 10.7759/cureus.94366

**Published:** 2025-10-11

**Authors:** Magdalena Alexieva, Evgeni V Mekov, Silvia Ivanova, Alexandrina Vlahova, Georgi Yankov

**Affiliations:** 1 Thoracic Surgery, University Hospital “St. Ivan Rilski”, Medical University of Sofia, Sofia, BGR; 2 Pulmonary Diseases, Medical University - Sofia, Sofia, BGR; 3 Pathology, University Hospital “St. Ivan Rilski”, Medical University of Sofia, Sofia, BGR; 4 Pathology, Medical University - Sofia, Sofia, BGR

**Keywords:** benign tumor, peripheral nerve sheath tumor, resection, retroperitoneal tumors, schwannoma, surgery

## Abstract

Retroperitoneal schwannomas are rare benign tumors of peripheral nerve sheath origin. We report a 72-year-old woman presenting with abdominal pain, altered bowel habits, and intermittent fever. CT imaging revealed a large (83 × 83 × 104 mm), well-circumscribed retroperitoneal mass with calcifications and heterogeneous enhancement, without invasion of adjacent structures. Following multidisciplinary discussion among thoracic surgery, medical oncology, pathology, and radiation oncology, a midline laparotomy was performed, and the tumor was completely excised. Histology demonstrated Antoni A and B areas, cystic degeneration, and focal palisading. Immunohistochemistry was positive for SOX10 and negative for CD34, DOG1, and desmin, confirming schwannoma. The postoperative course was uneventful, and the patient was discharged on day 14. We also conducted a focused review.

## Introduction

Retroperitoneal tumors (RTs) are a rare and heterogeneous group of neoplasms, with an estimated incidence of 0.5-1.0 per 100,000 population, and retroperitoneal schwannomas account for only ~1-5% of RTs [1,2]. They are defined as tumors arising within the retroperitoneal space (including the presacral and pelvic floor regions) but excluding the solid retroperitoneal organs [1]. Primary RTs may arise from various tissue types, including adipose tissue, connective tissue, muscle, blood vessels, lymphatic tissue, nerves, and embryonic remnants [3].

Among the uncommon types of RTs are schwannomas (also known as neurilemmomas), which are benign nerve sheath tumors derived from Schwann cells that envelop peripheral nerves [4]. Clinically, presentations are nonspecific and largely determined by tumor size and location. Most retroperitoneal schwannomas remain asymptomatic until they become large - series describe diameters ranging from ~5 cm to >30 cm, at which point they may cause pain, fullness, or compressive symptoms [5]. Although certain radiologic features (such as a well-circumscribed encapsulated mass with cystic degeneration and heterogeneous enhancement) may suggest schwannoma, preoperative diagnosis is challenging because radiologic features overlap with other mesenchymal tumors (e.g., liposarcoma, leiomyosarcoma), and surgical planning can be complex due to the proximity of major vessels and vital organs within the retroperitoneum [6]. Histopathology and immunohistochemistry, particularly diffuse S-100 protein positivity, remain essential for definitive diagnosis [7]. Complete surgical excision is considered the treatment of choice, with recurrence being rare after adequate resection [8].

In this report, we present a rare case of a retroperitoneal schwannoma successfully treated with surgical resection, accompanied by a brief review of the relevant literature.

## Case presentation

In May 2025, a 72-year-old woman was admitted to the department of thoracic surgery with a one-month history of gradually progressive abdominal pain, alternating constipation and diarrhea, and intermittent low-grade fever. Her medical history included hypertension, glaucoma, and nodular goiter. She had no known risk factors. Physical examination revealed no palpable abdominal mass and no abdominal tenderness, and laboratory studies were within normal limits.

**Figure 1:**
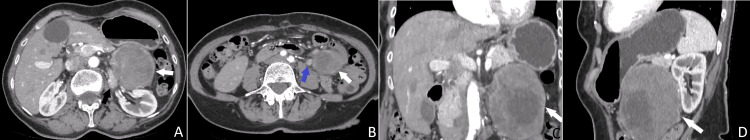
Computed tomography images of the retroperitoneal schwannoma. A, B. axial views. C. coronal view. D. sagittal view. The primary tumor is indicated by a white arrow. A second cranio-medial tumor is marked with a black arrow, and a smaller caudal tumor is marked with a blue arrow.

Computed tomography (CT) revealed an oval, well-circumscribed mass located in the left middle abdominal quadrant, measuring 83 × 83 × 104 mm (Figure 1). The mass had smooth, well-defined borders and was encapsulated by a contrast-enhancing rim containing calcifications. Its internal structure was heterogeneous, with gradual contrast enhancement, most pronounced during the venous phase. Anatomically, the mass was in proximity to the stomach and the tail of the pancreas cranially, the psoas muscle medially, the small intestine ventrally, and caudally, and it exerted compressive effects on the left kidney dorsally. There were no radiological signs of invasion into adjacent organs, and the surrounding fat planes remained preserved. Based on these features, the initial differential included retroperitoneal sarcoma (e.g., liposarcoma or leiomyosarcoma), gastrointestinal stromal tumor, paraganglioma, and a neurogenic tumor.

 Additionally, two contrast-enhancing irregular oval structures near the tumor, one measuring 23 × 12 mm cranially and medially, and a smaller lesion caudally near the lower pole, were confirmed intraoperatively as para-aortic lymph nodes and were excised. Hepatomegaly and multiple liver cysts were noted as incidental findings. Based on imaging characteristics, the tumor was considered most consistent with a mesenchymal neoplasm. The differential diagnosis included gastrointestinal stromal tumor (GIST), schwannoma, and sarcoma.

A fibrocolonoscopy revealed intestinal diverticulosis and fourth-degree hemorrhoidal disease. The case was reviewed in a multidisciplinary team meeting involving thoracic surgery, medical oncology, pathology, and radiation oncology, and surgical intervention was recommended.

**Figure 2:**
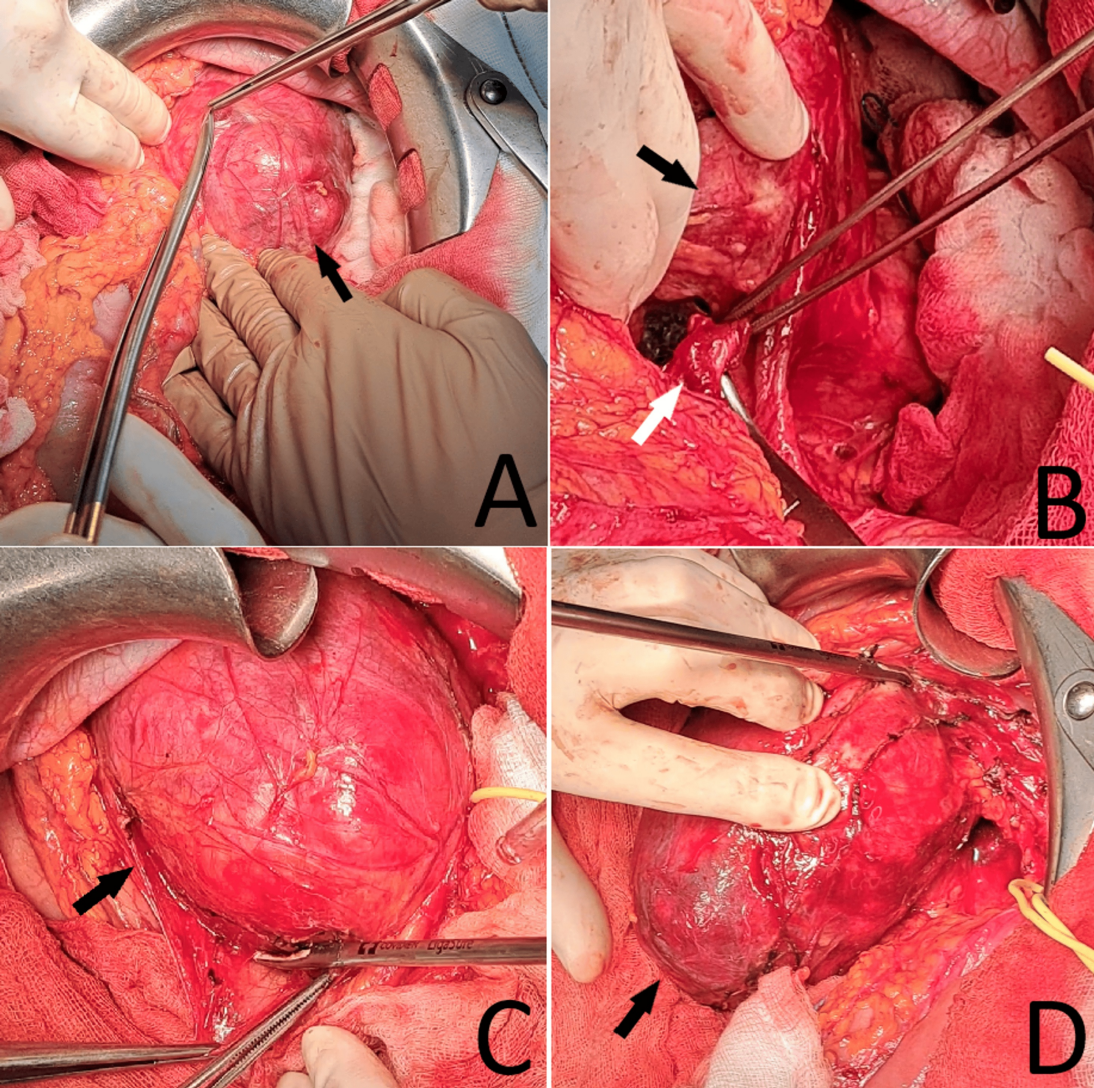
Intraoperative photographs. A–D: stepwise dissection of the retroperitoneal schwannoma. The tumor is marked with a black arrow; the ureter is marked with a white arrow.

​​​​​​A midline xiphoid-pubic laparotomy was performed. Dense adhesions were found between the transverse and descending colon, necessitating debridement. A firm, elastic mass was palpated anterior to the left kidney and the proximal ureter, lateral to the abdominal aorta, and inferior to the pancreatic body (Figure 2).

The left colic flexure was mobilized, and the left portion of the gastrocolic ligament was incised using a Harmonic scalpel. The flexure and the left colon were retracted medially. The left ureter, along with its accompanying vessels, the left ovarian artery and vein, and the left renal vein, were carefully dissected and safely dissected free.

The tumor, measuring approximately 100 × 130 mm, was mobilized and excised. A diagnostic para-aortic lymphadenectomy was performed, with intraoperative frozen-section analysis of the primary mass favoring a mesenchymal neoplasm and frozen-section evaluation of a sampled para-aortic node showing no tumor cells.

**Figure 3:**
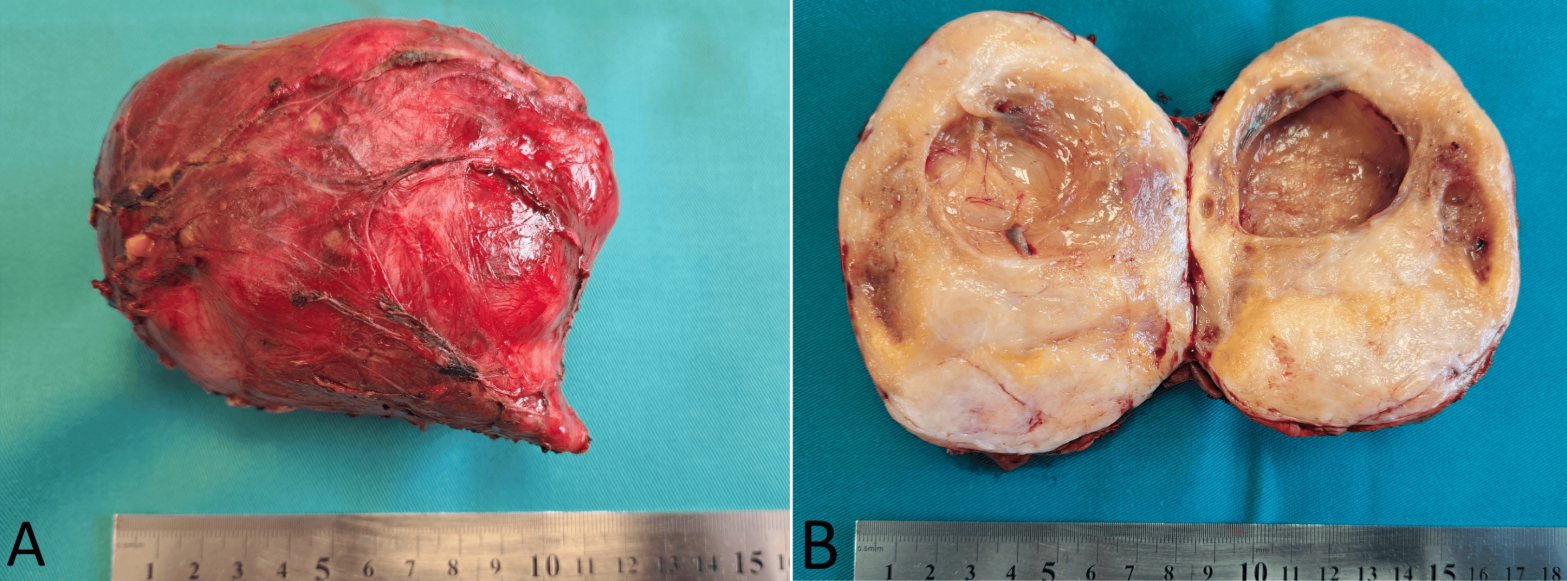
Postoperative specimen of the retroperitoneal schwannoma. A. intact tumor. B. dissected tumor.

Macroscopically, the tumor was well demarcated and rounded, encased by a smooth, thick fibrous capsule, and on sectioning showed a tan-yellow cut surface predominantly solid with central cystic degeneration, without gross hemorrhage, necrosis, or calcifications, measuring 105 × 85 mm (Figure 3).

**Figure 4:**
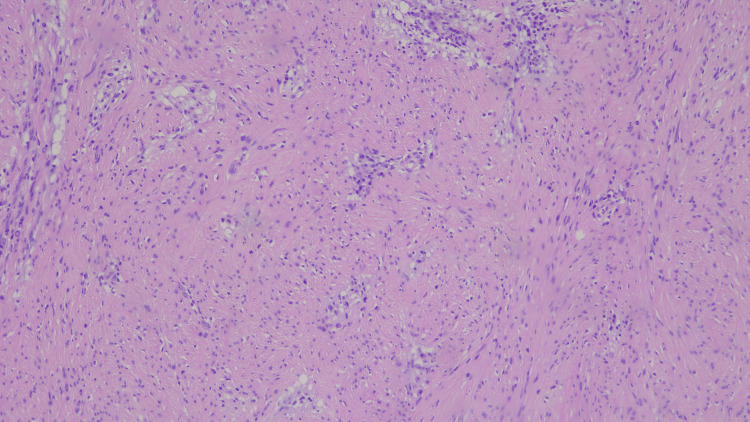
Histological image of the schwannoma. Hematoxylin and eosin staining, magnification 20×40.

Histopathological examination confirmed a mesenchymal neoplasm characterized by scattered intra- and peritumoral lymphoplasmacytic infiltrates, some forming perivascular cuffing or perivascular patterns. The tumor demonstrated classic Antoni A (hypercellular) and Antoni B (hypocellular) areas composed of spindle-shaped cells with eosinophilic cytoplasm, with regions of cystic degeneration and focal palisading (Figure 4). No areas of necrosis were identified, and the mitotic index was very low (<1/10 HPF).

**Figure 5:**
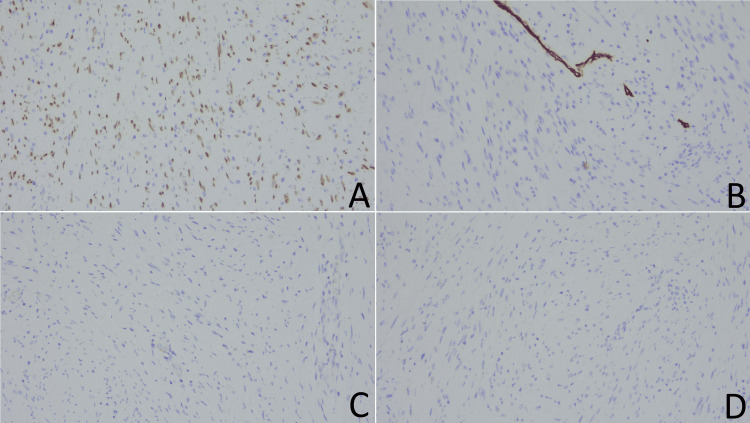
Immunohistochemical analysis. A. positive nuclear staining for SOX10. B–D. negative staining for CD34 (B), DOG1 (C), and desmin (D).

Immunohistochemical analysis revealed a positive nuclear reaction for SOX10 and negative staining for CD34, DOG1, and desmin (Figure 5). The immunomorphological findings were consistent with a peripheral nerve sheath tumor - schwannoma.

The patient was discharged on postoperative day 14 without complications. At the three-month follow-up, she was asymptomatic and underwent abdominal ultrasonography. Contrast-enhanced CT and MRI of the abdomen are scheduled at six months.

## Discussion

Schwannomas are rare, typically benign neoplasms of peripheral nerve sheath origin, most often presenting as solitary, encapsulated masses attached to adjacent tissues or organs [9]. Clinical manifestations are generally nonspecific and depend on tumor size and location, with abdominal discomfort and neurological deficits being the most common [4]. Notably, our patient presented with altered bowel habits and intermittent fever, which are less typical. Retroperitoneal localization is particularly uncommon, accounting for only 1-3% of all schwannomas [4], and these tumors are often detected incidentally due to their deep location and slow growth. Reported tumors with diameters ranging from 5 to 300 mm have shown a mean growth rate of 1.9 mm/year (range: 1.9-8.7 mm/year) [10,11]. Rapid growth should raise suspicion for malignant transformation or other aggressive tumors, such as leiomyosarcomas, undifferentiated liposarcomas, or malignant peripheral nerve sheath tumors [11].

The majority of retroperitoneal tumors are malignant and can be broadly categorized into solid and cystic types [12]. Retroperitoneal schwannomas are usually benign and solitary but may present as multiple tumors in patients with neurofibromatosis type 2 [13]. They may display either infiltrative or expansive growth patterns. Imaging-based criteria to differentiate benign from malignant retroperitoneal tumors include ill-defined margins, irregular surface, size thresholds (major diameter >5.85 cm, minor diameter >5.35 cm), solid texture, and an apparent diffusion coefficient <1.2 × 10⁻³ mm²/s, with varying sensitivity (36.0-80.0%) and specificity (58.1-93.5%) [14]. Correct identification is essential to exclude malignant and other benign differentials such as metastatic adenopathy, cystic lymphangioma, paraganglioma, and gastrointestinal stromal tumors [15].

CT and magnetic resonance imaging (MRI) are the primary diagnostic modalities. Retroperitoneal schwannomas typically appear as well-circumscribed, encapsulated masses, with larger tumors demonstrating heterogeneous enhancement from cystic degeneration and occasional curvilinear or punctate calcifications, while MRI features such as central enhancement and the “target sign” support a benign schwannoma [13,11]. Despite occasional atypical features, benign retroperitoneal schwannomas rarely metastasize or recur after complete excision [10].

Although preoperative biopsy (Tru-Cut or fine-needle aspiration) can aid diagnosis, it was not performed in our case because surgery had already been recommended and would provide both definitive diagnosis and therapy without delaying treatment. Real-time image-guided retroperitoneal puncture biopsy, including fusion imaging with an 18F-fluorodeoxyglucose positron emission tomography / computed tomography (18F-FDG PET/CT), has proven to be a safe and accurate approach [1]. Retroperitoneoscopic biopsy offers another minimally invasive alternative for both benign and malignant tumors [16]. In the present case, preoperative biopsy was not performed due to strong surgical indications with a high clinical suspicion of sarcoma.

Histologically, schwannomas consist of two classic components: densely cellular Antoni type A areas and loosely arranged and hypocellular Antoni type B areas with microcystic changes [8]. Malignant forms demonstrate atypia, ulceration, and invasive growth patterns [10]. Immunohistochemically, schwannomas consistently express S100, SOX10, and vimentin, which are useful in establishing the diagnosis [10]. These hallmarks help distinguish benign schwannoma from malignant differentials such as malignant peripheral nerve sheath tumor, characterized by reduced/heterogeneous S100/SOX10 expression, higher mitotic activity, and necrosis, and from non-neurogenic sarcomas [e.g., leiomyosarcoma, smooth muscle actin (SMA)/desmin positive or gastrointestinal stromal tumor (GIST) c-KIT/CD117 (KIT)/ Discovered On GIST-1 (DOG1) positive].

While a “wait-and-watch” approach may be considered for small, asymptomatic tumors due to their indolent nature [11], observation should include imaging surveillance (MRI or CT every 6-12 months for the first 1-2 years, then annually if stable), whereas complete surgical resection remains the only potentially curative treatment [4]. These tumors are typically resistant to radiotherapy and chemotherapy [13]. Surgical approach depends on tumor size, location, and relationship to adjacent structures. Large tumors closely associated with major vessels or organs may not be suitable for laparoscopic removal [17], whereas small, well-circumscribed tumors can often be safely excised laparoscopically [17,18]. Case reports describe successful resection of giant retroperitoneal schwannomas up to 20 × 15 × 10 cm [9] and even multiple interconnected tumors [17]. In our patient, a total midline laparotomy with debridement and complete excision of the left retroperitoneal mass was performed. Given the tumor’s large size and close relationship to vital retroperitoneal structures, an open surgical approach was favored over laparoscopy.

## Conclusions

Retroperitoneal schwannoma is rare and diagnostically challenging because presenting symptoms are nonspecific and imaging features overlap with other retroperitoneal masses. This case underscores the importance of integrating imaging, histopathological confirmation, and multidisciplinary surgical planning to secure an accurate diagnosis and guide management. When surgery is already indicated, primary complete excision provides both definitive diagnosis and curative treatment. Prognosis after R0 resection is excellent, with periodic imaging follow-up advised.
